# Genome-wide identification, evolution and transcriptome analysis of GRAS gene family in Chinese chestnut (*Castanea mollissima*)

**DOI:** 10.3389/fgene.2022.1080759

**Published:** 2023-01-04

**Authors:** Liyang Yu, Cai Hui, Ruimin Huang, Dongsheng Wang, Cao Fei, Chunlei Guo, Jingzheng Zhang

**Affiliations:** ^1^ Engineering Research Center of Chestnut Industry Technology, Ministry of Education, Hebei Normal University of Science and Technology, Qinhuangdao, Hebei, China; ^2^ Hebei Collaborative Innovation Center of Chestnut Industry, Qinhuangdao, Hebei, China; ^3^ The Office of Scientific Research, Hebei Normal University of Science and Technology, Qinhuangdao, Hebei, China; ^4^ Hebei Key Laboratory of Horticultural Germplasm Excavation and Innovative Utilization, Qinhuangdao, Hebei, China

**Keywords:** *Castanea mollissima*, GRAS family, duplication model, expression pattern, protein structure

## Abstract

GRAS transcription factors play an important role in regulating various biological processes in plant growth and development. However, their characterization and potential function are still vague in Chinese chestnut (*Castanea mollissima*), an important nut with rich nutrition and high economic value. In this study, 48 *CmGRAS* genes were identified in Chinese chestnut genome and phylogenetic analysis divided *CmGRAS* genes into nine subfamilies, and each of them has distinct conserved structure domain and features. Genomic organization revealed that *CmGRAS* tend to have a representative GRAS domain and fewer introns. Tandem duplication had the greatest contribution for the *CmGRAS* expansion based on the comparative genome analysis, and *CmGRAS* genes experienced strong purifying selection pressure based on the *Ka/Ks*. Gene expression analysis revealed some *CmGRAS* members with potential functions in bud development and ovule fertility. *CmGRAS* genes with more homologous relationships with reference species had more *cis*-acting elements and higher expression levels. Notably, the lack of DELLA domain in members of the DELLA subfamily may cause de functionalization, and the differences between the three-dimensional structures of them were exhibited. This comprehensive study provides theoretical and practical basis for future research on the evolution and function of GRAS gene family.

## Introduction

GRAS proteins are an important family of plant-specific transcription factors and extensively exist in plant, whose name was derived from the three originally functionally characterized genes, GAI, RGA and SCR (Pysh et al., 1999; Khan et al., 2022). GRAS family genes received extensive attention a long time ago, known as “green revolution” genes (Peng et al., 1999). Specifically, world wheat grain yields increased substantially in the 1960s and 1970s because farmers rapidly adopted the new varieties of the so-called “green revolution”. The new varieties are shorter, increase grain yield at the expense of straw biomass, and are more resistant to damage by wind and rain. The reason why these new varieties are shorter is that they respond abnormally to the plant growth hormone gibberellin (Börner et al., 1996). Subsequent studies showed that this reduced response to gibberellin was caused by mutations two *Reduced height-1* (*Rht-B1* and *Rht-D1*) loci, and they are the orthologues of the *Arabidopsis Gibberellin Insensitive* (*GAI*) gene (Winkler and Freeling, 1994; Peng et al., 1997). Actually, *GRAS* genes are involved in many biological processes in plant growth and development, such as signal transduction and response to stresses (Benfey et al., 1993; Di Laurenzio et al., 1996; Helariutta et al., 2000; Shahan et al., 2022). GRAS proteins are generally composed of 400–770 amino acids, and their C-terminal contains five highly conserved domains including LHRI, VHIID, LRII, PFYRE and SAW. The N-terminal sequences are diverse and participate in different signal transduction pathways, which determines the specificity of their functions (Sun et al., 2011).

*GRAS* genes have been genome-wide characterized in a large number of species, such as tomato (*Solanum lycopersicum*) and Chinese cabbage (*Brassica rapa*), which had classified the *GRAS* genes into eight subfamilies (Song et al., 2014; Huang et al., 2015). However, *GRAS* genes are divided into more subfamilies in some species, such as 13 subfamilies in *Populus trichocarpa* (Liu and Widmer, 2014), 16 in *Medicago truncatula* (Song et al., 2017), and 17 in some angiosperms (Cenci and Rouard, 2017), which implied that new subfamilies may be identified in plants without *GRAS* gene analysis (Yu et al., 2021). In addition, previous studies have reported multiple functions of GRAS gene family, such as root radial patterning, signal transduction, axillary meristem formation, gametogenesis and stress response (Khan et al., 2022). It has been found that at least 23 *GRAS* members play roles in the gibberellins (GAs) signal transduction pathway in *Arabidopsis thaliana*, which have high sequence similarity and participate in the regulation of GA at the N-terminal, and most of them belong to the DELLA subfamily (Gubler et al., 2002; Griffiths et al., 2006; Heo et al., 2011). The *SlLS* gene, a member of GRAS family in tomato, is involved in the process of the formation of lateral buds from the meristem of the leaf axil, and its mutation caused the cell vegetative growth stage of the leaf axil primordium to lose its ability to regenerate (Gubler et al., 2002). In addition, A large number of GRAS family members can respond to a variety of biotic and abiotic stresses. *OsCIGR2* gene in rice (*Oryza sativa*) can be used as a transcription activator to inhibit the death of rice cells infected by pathogenic bacteria by regulating the expression of *OsHsf23* (Tanabe et al., 2016). *VaPAT1* is a new member of PAT1 subfamily in grape (*Vitis vinifera*), and its overexpression in *A. thaliana* can improve drought resistance, cold resistance and salt tolerance (Yuan et al., 2016). The functions of some members of GRAS gene family have been characterized in model plants, but their potential functions in more species still need to be further studied (Yu et al., 2021).

*C. mollissima* BL (Chinese chestnut) is not only an important food resource in the northern hemisphere, but also is one of the earliest fruit trees domesticated in ancient China (Nie et al., 2021; Zhou et al., 2021). As early as 6,000 years ago, Chinese began to eat Chinese chestnuts fruit (Nie et al., 2021). Chinese chestnut has been regarded as an important ecological and economic tree species due to its rich nutrition, disease resistance and barren resistance (Chen et al., 2019). At present, China’s output of Chinese chestnut has been ranking first in the world for a long time, and the nut yield of Chinese chestnut of China is approximately 1.6 million tons, which accounted 83.3% of the world’s total production in 2020 (http://www.fao.org/home/en/) (Nie et al., 2021). Besides the high nutritional and ecological value, Chinese chestnut also has important medicinal value. Chinese chestnuts are rich in a variety of acidic amino acids (such as aspartic acid and glutamic acid), which can provide protons to quench unpaired electrons and radicals, thereby protecting human cells from harm (Zou et al., 2016; Feng et al., 2018). Glutamate can also participate in brain protein metabolism and improve central nervous system activity (Nomura et al., 2000). Chinese chestnut is rich in glycine, which can control blood cholesterol and blood sugar level to prevent and treat hypertension and diabetes. In addition, the rich calcium, iron, zinc and selenium in Chinese chestnut fruits also play important role in maintaining human health (Nomura et al., 2000; Dragicevic et al., 2015; Tomasi et al., 2015).

This study comprehensively characterized the GRAS transcription factors of Chinese chestnut at the whole-genome level for the first time, and analyzed their physicochemical properties, phylogenetic tree, conserved structure, duplication models, transcriptome of buds and fertile/sterile ovules at different stages. These analyses provide a reference for revealing the evolution and potential functions of *GRAS* genes in Chinese chestnut.

## Materials and methods

### Identification and GO/KEGG enrichment analysis of *CmGRAS* genes

The Chinese chestnut and rice genome information were obtained from Castanea Genome Database (http://castaneadb.net/) and RGAP (http://rice.plantbiology.msu.edu), respectively. The *A. thaliana* and grape genome data were obtained from Phytozome (phytozome.jgi.doe.gov/). The oak (*Quercus robur*) genome data were download from INRA (http://www.oakgenome.fr/). Three steps were used to identify the GRAS gene in Chinese chestnut. Firstly, thirty-two GRAS proteins in *A. thaliana* were used as BLAST queries to search against the Chinese chestnut protein datasets with default parameters (Supplementary Table S1). Next, the latest Hidden Markov Model (HMM) file corresponding to the GRAS domain (PF03514) was used to search against the output of the previous step with an E-value of 1e-5 using HMMER 3.0. Finally, the Batch-CDD was used to check the existence of the GRAS domain to finally confirm GRAS genes, and only members whose protein length was 350–820 aa were retained (Liu et al., 2018; Yu et al., 2021). In addition, we conducted a reciprocal BLAST search by using the proteins sequences of candidate *CmGRAS* genes as queries against *A. thaliana* GRAS proteins to verify the veracity of candidates (Supplementary Table S2). A total of 48 *CmGRAS* genes were identified, and their physicochemical properties, such as protein molecular weight (MW), isoelectric point (pI), aliphatic index, instability index, and grand average of hydropathicity (GRAVY) were obtained using tools from ExPasy website (Supplementary Table S3) (Artimo et al., 2012). We used eggNOG-mapper (http://eggnog-mapper.embl.de/) to obtain GO and KEGG annotation files of all Chinese chestnut genes, and TBtools was used for GO and KEGG enrichment analysis and visualization.

### Phylogenetic and sequence analysis

Considering that *A. thaliana* and rice were commonly used model plants to study genetic evolution, we used the ClusterW program to conduct multiple alignment of the full-length sequences of CmGRAS proteins (Supplementary Table S4). MEGA7.0 was used to obtain a phylogenetic tree using the maximum likelihood method. Specifically, the “Find Best DNA/Protein Models (ML)” function in MEGA7.0 was used to find the best amino acid substitution model (partial deletion 95%), and the final selection model was “JTT + G”. The final parameters were as follows: Jones–Taylor–Thornton (JTT) model; Gamma Distributed (G); Partial deletion 95%; 1,000 bootstrap replications. In addition, a phylogenetic tree was generated using neighbor-joining method of MEGA7.0 with the following parameters: Poisson model, pair-wise deletion, and 1,000 bootstrap replications. The GRAS gene family members in Chinese chestnut were divided into different subfamilies, whose names were derived from the recognized subfamily names of *A. thaliana GRAS* genes (Pysh et al., 1999; Liu et al., 2018). The phylogenetic tree of the Chinese chestnut *GRAS* genes was constructed for comprehensive analysis with gene structure, protein conserved domains and motifs. The MEME online program was used to identify motifs in Chinese chestnut GRAS proteins, and TBtools was used to obtain the exon-intron organization and intron phase of the *CmGRAS* gene from the structural annotation of the Chinese chestnut genome (Chen et al., 2020).

### Collinear analysis and *CmGRASs* duplication models

In order to further infer the phylogenetic mechanisms of Chinese chestnut GRAS gene family, the collinearity of Chinese chestnut, *A. thaliana*, rice, oak and grape genomes was obtained by Multiple Collinearity Scan toolkit (MCScanX) software (Wang et al., 2012). We performed further analysis to distinguish the duplication models of *CmGRAS* genes, as in our previous study (Yu et al., 2022). Briefly, we first analysed the complementary relationship between different homologous fragments by drawing the homologous collinearity dot-plots within the Chinese chestnut genome. Next, the “add_ka_and_ks_to_collinearity” in MCScanX was used to calculate the synonymous substitution sites (*Ks*) values of homologous genes on the homologous fragments, which help us to obtain the approximate age of the generation of different homologous fragments. Finally, we determined the *CmGRAS* gene from the whole-genome duplication (WGD) event based on the normally distributed *Ks* value corresponding to the eudicot common hexaploidization event (ECH) and the complementary relationship of the homologous fragments. Other duplication models were directly confirmed by MCScanX. The visualization of the collinearity relationship was obtained by TBtools (Chen et al., 2020), and the median *Ks* values was calculated by writing script (Yu et al., 2021).

### *Cis*-acting elements analysis of *CmGRASs*

Considering that large genomes usually had *cis*-acting elements as far as 10,000 bp, we obtained the upstream 10,000 bp sequence (promoter regions) of all 48 CmGRAS transcription initiation sequence from the Chinese chestnut genome to conduct *cis*-acting regulatory elements analysis. We submitted these sequences to the PlantCARE software to identify *cis*-acting elements and functional categorization. The *cis*-acting elements without knowing the specific function were removed, and TBtools was used to visualize the final results (Chen et al., 2020).

### Expression analysis of *CmGRAS* genes

Taking N11-1 as the reference genome (Castanea Genome Database), the gene expression data of Chinese chestnut were obtained from the National Center for Biotechnology Information (NCBI). RNA-seq data were obtained from two experiments. The first RNA-seq experiment measured the transcriptome changes of Chinese chestnut buds at different development stages (SRP389011). In the experiment, RNA samples of buds were collected at 20, 25 and 30 days after flowering, with three biological replicates. The Illumina NovaSeq 6000 platform were used to perform RNA sequencing to generate 100 bp pair-end reads. The Kallisto software was used to map the reads against the Chinese chestnut genome. TPM (Transcripts Per Kilobase of exon model per Million mapped reads) were calculated for each gene. The second RNA-seq experiment measured the transcriptome of fertile and abortive ovules sampled at different development stage (15-July, 20-July and 25-July) (SRP364950). The purpose of this study was to detect the genes affecting ovule fertility through transcriptome analysis of fertile and abortive ovules at different stages. RNA samples of fertile and abortive ovules were collected from three Chinese chestnut trees. RNA-seq was run using Illumina Hiseq 2000 to obtain 100 bp pair-end reads, and TPM of each gene were calculated. GRAS genes from two experiments were scanned to analyse their potential functions in bud development and ovule fertility. The “Normalized” (scale method) in TBtools was used to normalize the expression, and the heatmaps were created by TBtools based on the transformed data of log2 (FPKM+ 1) values (Chen et al., 2020).

### Three-dimensional structure analysis of *CmGRAS* proteins

*CmGRAS2* in DELLA subfamily can hardly detect transcripts in all samples by analysing RNA-seq data from two experiments. We constructed and compared the three-dimensional structure of the DELLA subfamily members to show and find more structural differences, besides the existence or not of the DELLA conserved domain. Specifically, we first obtained the three-dimensional structure of the four members of the DELLA subfamily through the Swiss model (https://swissmodel.expasy.org/). Next, we evaluated the reliability of the predicted three-dimensional structure, and the tools used were mainly including GMQE (Global Model Quality Estimate) and QMEANDisco Global in Swiss model, and three other protein structure reliability evaluation tools (ERRAT, Verify3D, PROCHECK) on the SAVES website (Lüthy et al., 1992; Colovos and Yeates, 1993; Laskowski et al., 1993). When the GMQE and QMEANDisCo Global assessment results were reliable (GMQE > 0.4 and QMEANDisCo Global > 0.6), we conducted other quality assessments on the SAVES website. Additionally, the Alphafold 2 was used to construct the three-dimensional structure of the four members of the DELLA subfamily with default parameters (Jumper et al., 2021). Visual Molecular Dynamics (VMD) (Version 1.9.4) was used to align the predicted three-dimensional structures of proteins, and the SuperPose Version 1.0 (Maiti et al., 2004) was used to calculate the similarity between three-dimensional structures.

## Results

### Identification of *CmGRAS* genes, physicochemical properties, sequence and phylogenetic analysis

**FIGURE 1:**
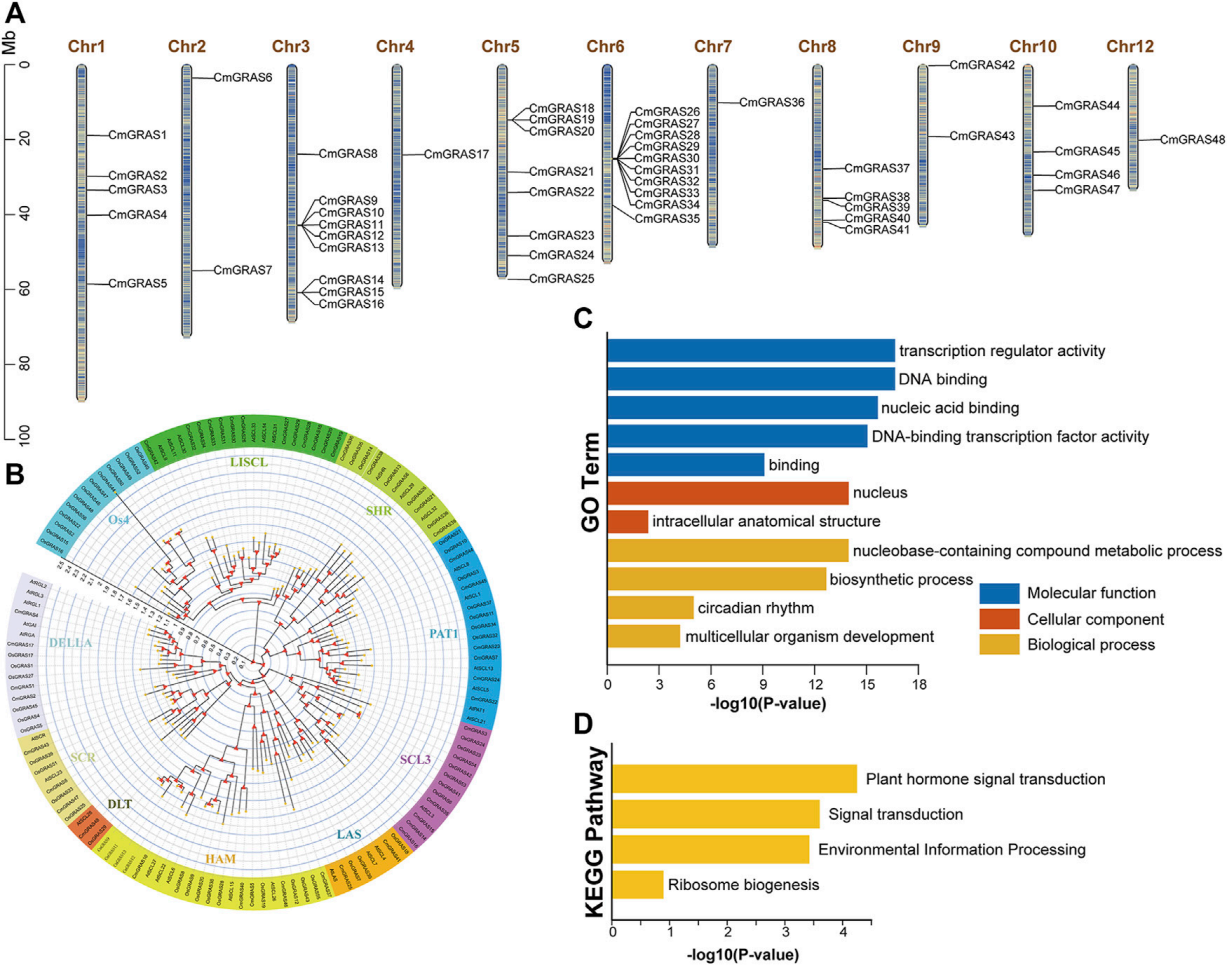
Chromosome distribution, phylogenetic analysis and functional enrichment analysis of *CmGRAS* genes. **(A)** Chromosome distribution of *CmGRAS* genes. The colour of segments in the chromosomes shows the gene density of the corresponding region. **(B)** The phylogenetic tree of *GRAS* genes in Chinese chestnut, *Arabidopsis thaliana* and rice was constructed by maximum likelihood method. **(C)** GO function enrichment analysis of *CmGRAS* genes. **(D)** KEGG function enrichment analysis of *CmGRAS* genes.

We combined sequence similarity and conserved domain analysis to identify the *GRAS* gene in Chinese chestnut. A total of 48 *GRAS* gene family members in Chinese chestnut were identified, and they were renamed from *CmGRAS1* to *CmGRAS48* based on their position on the chromosomes (Figure 1A; Supplementary Tables S3). The *CmGRAS* genes were distributed on all chromosomes except Chr11. The largest number of GRAS genes was mapped to Chr6, followed by Chr3 and there were only one *CmGRAS* genes on Chr7. Notably, only a few *CmGRAS* members were located at both ends of chromosomes.

The physicochemical properties of CmGRAS proteins vary widely, ranging in molecular weight from 41.3 to 87.9 KDa and in length from 365 to 805 aa. The average theoretical isoelectric point (pI) was 5.85, implying that most HrGRAS proteins were weakly acidic. Only CmGRAS45 was considered as stable, duo to its instability index less than 40, and others were predicted as unstable. Except for CmGRAS16 and CmGRAS39, other CmGRAS proteins were predicted to be hydrophilic, because their GRAVY values were less than zero. Most CmGRAS proteins included many aliphatic amino acids, and the aliphatic indices of CmGRAS proteins range from 69.45 to 103.72. Subcellular localization predictions indicated that 30 *CmGRAS* members were thought to be located in the nucleus, 10 members were located in cytoplasmic, and others were located in plasma membrane and mitochondrial (Supplementary Table S3).

We performed phylogenetic analysis of 136 GRAS proteins from Chinese chestnut (48), *A. thaliana* (32) and rice (56) to understand the evolutionary relationship and taxonomy of CmGRAS (Figure 1B; Supplementary Figure 1). The result shows that all of CmGRAS proteins were classified into nine distinct subfamilies, and they termed as DELLA, PAT1, SCL3, SHR, SCR, LISCL, HAM, LAS and DLT. Os4 subfamily was considered unique to rice in previous studies, there were no CmGRAS protein. *CmGRAS* in the same clade as specific members in *A. thaliana* may have similar functions. For example, *CmGRAS41* belongs to LAS subfamily, so it is speculated that it may have similar functions with *AtLAS* members belonging to this subfamily, such as promoting the development of lateral meristem. Actually, the subsequent transcriptome analysis also found that *CmGRAS41* was consistently overexpressed throughout the bud development stages, and its expression in fertile ovules was significantly higher than that in abortive ovules.

The potential functions of all *CmGRAS* genes were described through annotation information from the GO and KEGG databases (Figures 1C,D). *CmGRAS* mainly enriched in the terms of transcription regulator activity and DNA-binding transcription factor activity, which were closely related to transcription factors. The KEGG enrichment result implied that they were involved in plant hormone signal transduction and environmental information processing, and these were consistent with previous evidence in other species that *GRAS* genes were involved in signal transduction, growth and development, biotic and abiotic stress.

### The motif distribution and gene structures of *CmGRASs*

**FIGURE 2:**
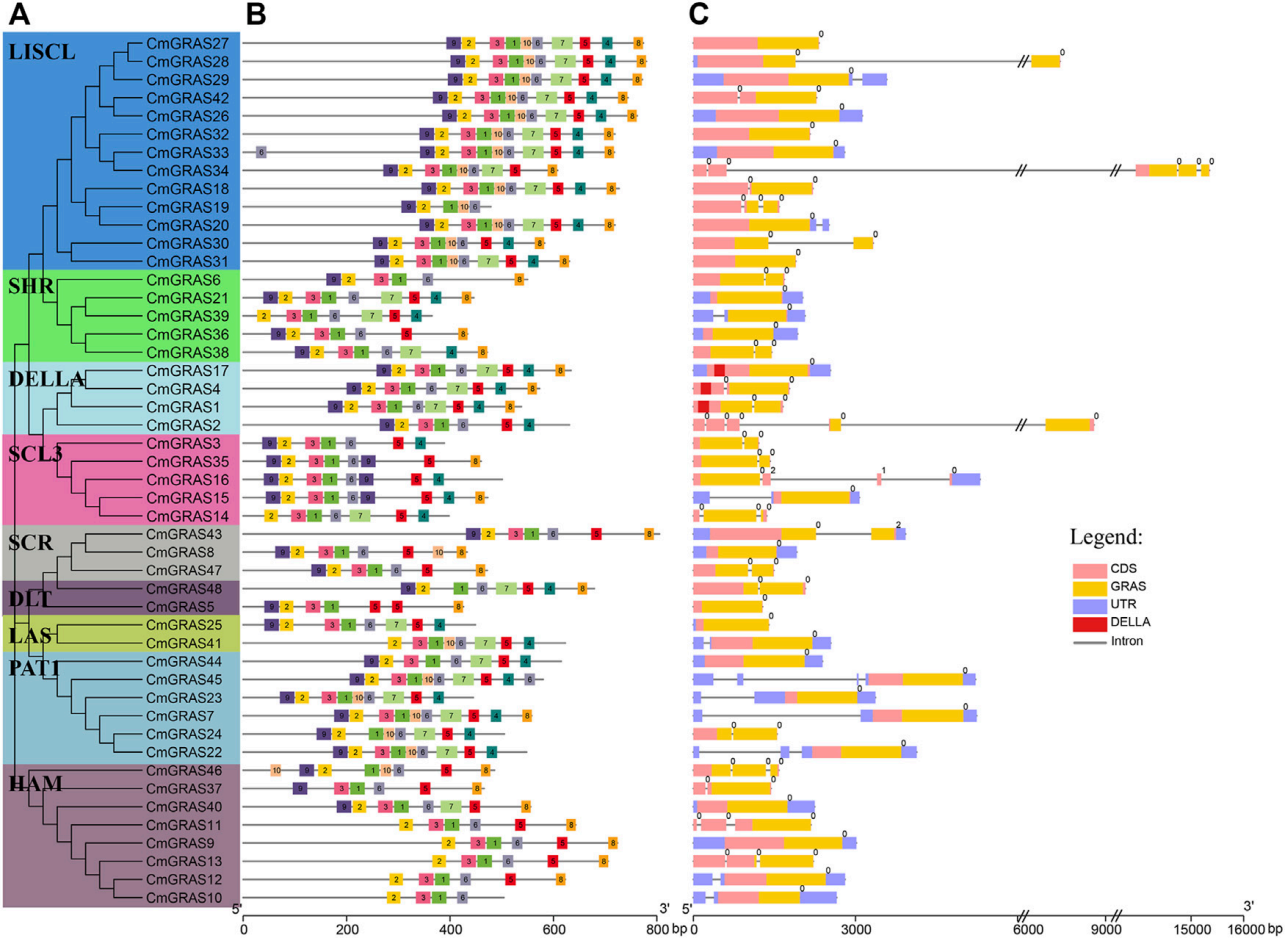
Phylogenetic tree, distribution of conserved motifs and gene structure of *CmGRAS* genes. **(A)** Phylogenetic analysis of *CmGRAS* genes. **(B)** Distribution of conserved motifs of *CmGRAS* genes. **(C)** Gene structure of *CmGRAS* genes.

The genetic characteristics of *CmGRAS* genes were analyzed to reveal their evolution (Figures 2A–C). As showed in Figure 2C, most *CmGRAS* genes included few introns (0 or 1), similar to the *GRAS* gene in other species, such as *A. thaliana*, tomato and seabuckthorn (*Hippophae rhamnoides*) (Liu and Widmer, 2014; Huang et al., 2015; Yu et al., 2021), considering that all Chinese chestnut genes contain four introns on average. Almost all introns of *CmGRAS* genes were phase zero, which implied they were extreme conserved. All *CmGRAS* members had one or more GRAS conserved domains, and the members belonging to the same subfamily also different in their conserved domains. For example, *CmGRAS2* belong to the DELLA subfamily, but it lacked the DELLA domain, which implied that it may loss DELLA conserved domain during evolution, but retained other features of the structure group.

We used MEME program to identify the *CmGRAS* genes motifs to further investigate their common feature. A total of 10 conserved motifs were identified, and most of them were widely distributed in the *CmGRAS* genes (Figure 2B). The motif arrangement pattern was almost the same as the result of phylogenetic analysis. Members in the same subfamily usually had similar motifs, but there were also some members of the subfamily with different motif distributions. For example, motif 1, 2, 3, 4 and 6 existed in all members of SCL3 subfamily especially their motif arrangements were similar. The motif arrangement of some members was exactly the same, such as *CmGRAS27/28/29*, *CmGRAS12/13*, which were all formed by tandem duplication. Contrastingly, compared with other members of HAM subfamily, *CmGRAS33* obviously had one more motif 6 at the N-terminal. It was worth noting that some members of the DELLA subfamily had exactly the same motif arrangement as the members of the SCL3 subfamily, but some members of the SCL3 subfamily had completely different motifs composition. These results suggested that the non-motif sequences of some *CmGRAS* genes had undergone changes in during evolution, forming different subgroups, but their motif composition was relatively conservative. Interestingly, the *CmGRAS2* was the only member of the DELLA subfamily that lacked both motif 7/8 and the DELLA conserved domain, prompting us to speculate that motif 7/8 may be an important component of the DELLA conserved domain. The roles of these conserved motifs required further studies to clarify their potential functions for plant growth.

### Collinear analysis of *CmGRASs*

**FIGURE 3:**
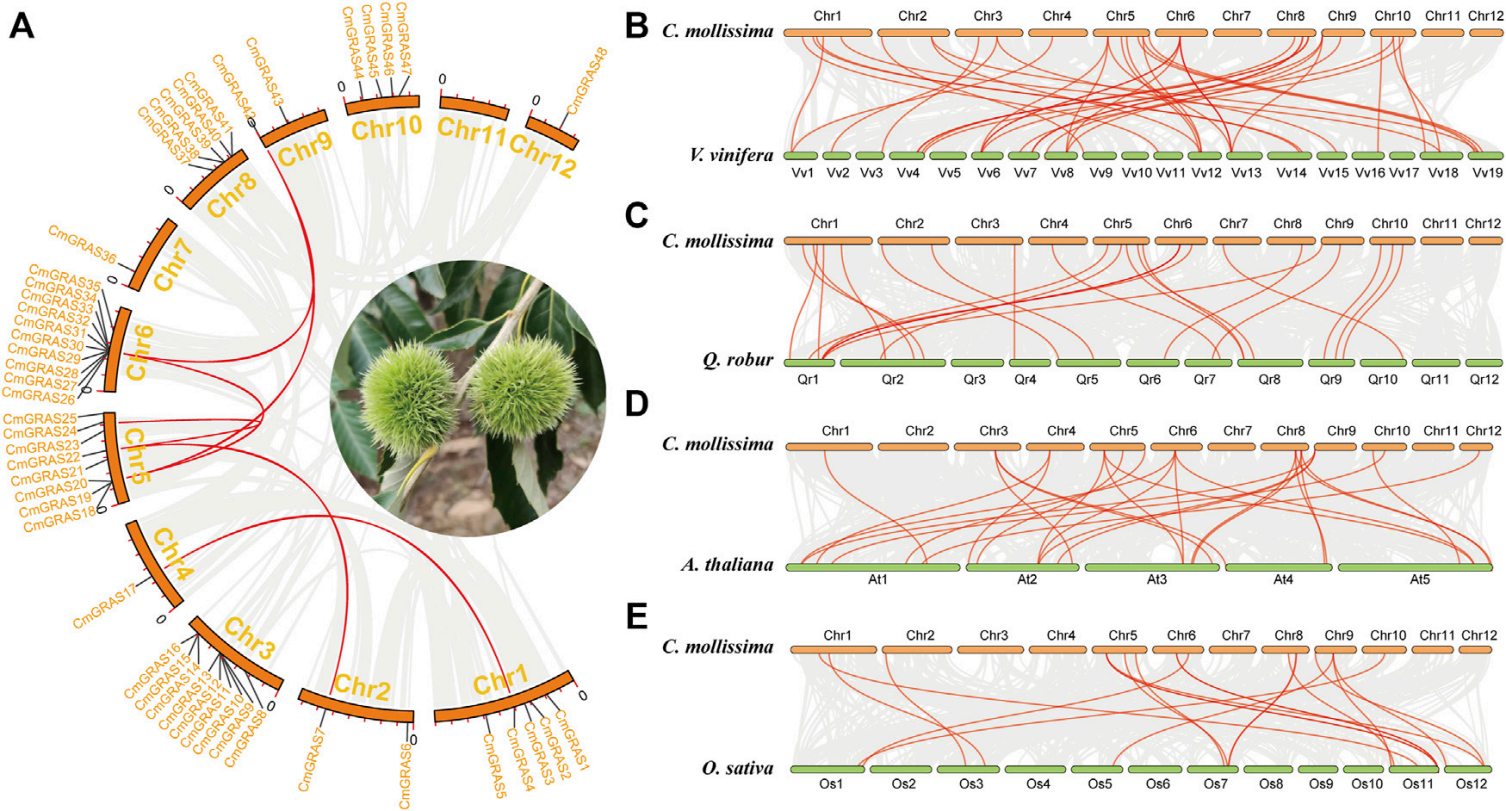
Collinear relationships within Chinese chestnut genome and with the grape, oak, *Arabidopsis thaliana* and rice genomes. **(A)** Collinear relationship within Chinese chestnut genome. **(B)** Collinear relationship between Chinese chestnut and grape genomes. **(C)** Collinear relationship between Chinese chestnut and oak genomes. **(D)** Collinear relationship between Chinese chestnut and *Arabidopsis thaliana* genomes. **(E)** Collinear relationship between Chinese chestnut and rice genomes.

Collinear relationships within the Chinese chestnut genome and with the *A. thaliana*, rice, grape and oak genomes were analyzed to explore the evolution of the *CmGRAS* genes (Figure 3). Most of the *CmGRAS* genes were not detected in collinear regions within the Chinese chestnut genome, suggesting that they were more active in evolution (Figure 3A) (Yu et al., 2021). The genomes of Chinese chestnut and grape, Chinese chestnut and oak, had 31 and 25 *CmGRAS* genes in the collinear region, respectively (Supplementary Tables S5, S6). There were only 16 *CmGRAS* genes in the collinear region between Chinese chestnut and *A. thaliana*, Chinese chestnut and rice (Supplementary Tables S7, S8). These results may be related to the fact that Chinese chestnut, oak and grape have not undergone other WGD event after the eudicot common hexaploidization event (ECH), which can make their genomes less affected by chromosome rearrangement after WGD.

Significantly, forty-one orthologous gene pairs including *CmGRAS* genes were identified between Chinese chestnut and grape, and this number was 25, 21 and 19 between Chinese chestnut and oak, Chinese chestnut and *A. thaliana*, Chinese chestnut and rice, respectively (Figures 3B–E; Supplementary Tables S5–S8). Some *CmGRAS* members were associated with more than three collinear gene pairs, such as *CmGRAS8*, *CmGRAS18*, *CmGRAS26*, *CmGRAS42* and *CmGRAS43*, which indicated that their orthologous genes were more retained in the *A. thaliana*, rice, oak and grape genomes. Based on the gene balance hypothesis (Birchler and Veitia, 2007; Xu et al., 2016; Yu et al., 2021), they may have important roles in the evolution of the CmGRAS gene family. Actually, subsequent analysis of transcriptome sequencing data also supported that they had an important role in the development of bud and ovule. In addition, the length of the collinear blocks including the *CmGRAS* collinear gene pairs between the Chinese chestnut and grape genomes (average of 56 gene pairs) (Supplementary Table S9) was much longer than that between Chinese *chestnut* and *A. thaliana* (average of 22 gene pairs) (Supplementary Table S10), between Chinese chestnut and rice (average of 14 gene pairs) (Supplementary Table S11) and between Chinese chestnut and oak (average of 14 gene pairs) (Supplementary Table S12). These statistical differences may be related to the phylogenetic relationship among species, the whole-genome duplication (WGD) event and the quality of genome assembly. In particular, it should be noted that the WGD was usually accompanied by a large number of chromosomal rearrangements, which could disrupt the detection of collinearity (Yu et al., 2022). Grape is a species with a relatively stable structure among core eudicots, and well preserved the chromosome structure formed by ECH event, which can explain the above statistical differences to some extent (Sato et al., 2012).

**FIGURE 4:**
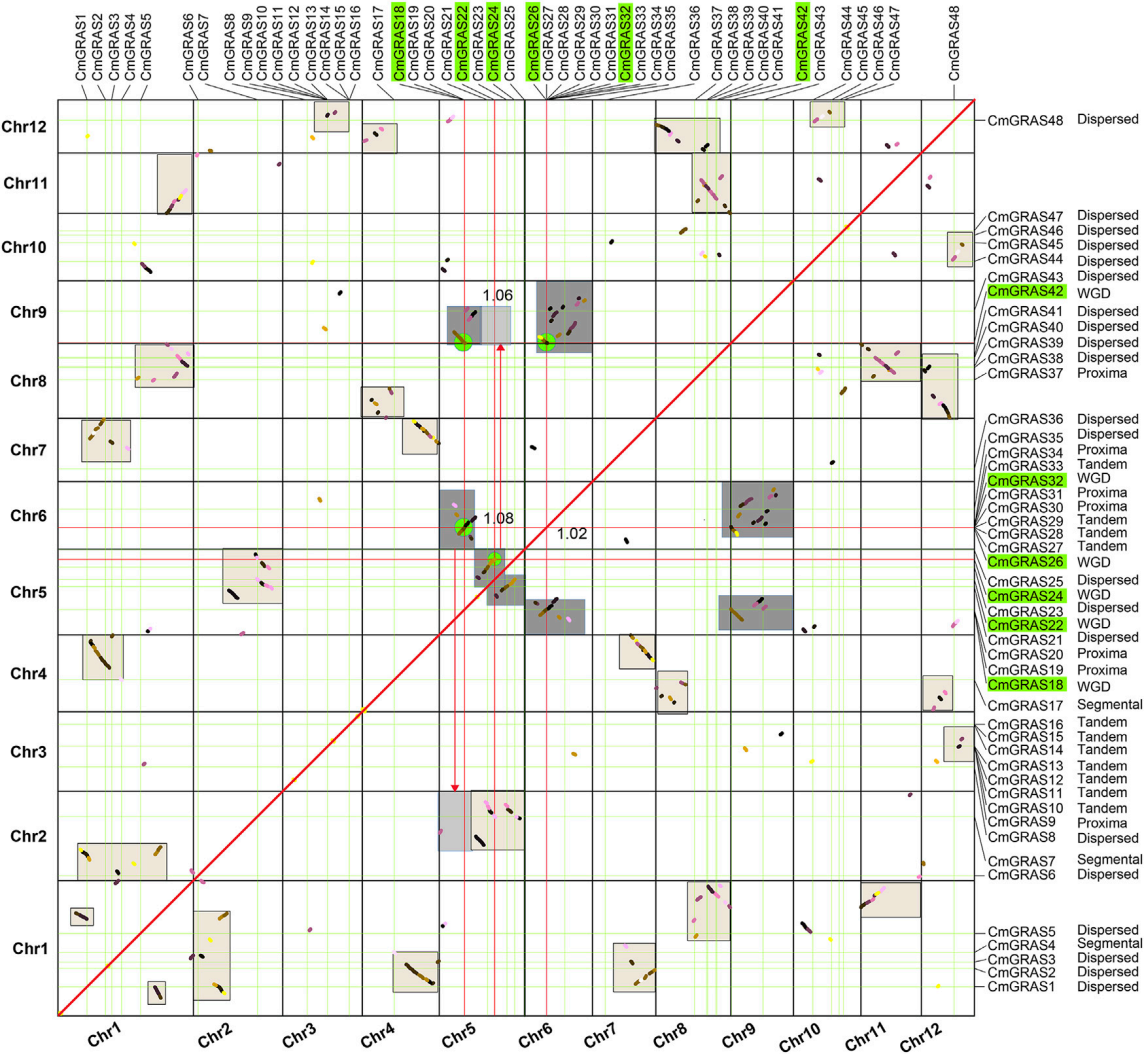
Homologous collinearity dot-plot within Chinese chestnut genome. The collinear blocks from WGD containing *CmGRAS* genes are marked in the gray boxes of the figure, and the median *Ks* of the collinear blocks are marked.

Gene duplication events had important contributions to the expansion of gene families, and to a certain extent affected the evolution of protein-coding gene families (Liu and Widmer, 2014; Liu et al., 2018; Quan et al., 2019). Multiple Collinearity Scan toolkit (MCScanX) program was performed to assign all *CmGRAS* genes to the four duplication types of WGD or segmental, proximal, tandem and dispersed. However, WGD was accompanied by a large number of fragmentation and fusion of chromosomes, especially which usually occurred millions of years ago. These variations at the chromosome level had caused confusion about whether the related genes originate from WGD or segmental duplication (Yu et al., 2022). Gaussian fitting was performed based on the median *Ks* of the collinear blocks in the chestnut genome to obtain the normal distribution corresponding to the ECH event (Supplementary Figure S2), as in our previous study (Yu et al., 2022), and the median *Ks* of the blocks where *CmGRAS* gene was located on the homologous dot-plot of Chinese chestnut genome were marked. Based on the median *Ks* of the homologous blocks, the homologous blocks lengths, and the complementary relationship between homologous blocks, we further distinguished *CmGRAS* members originated from WGD and segmental duplication (Figure 4; Supplementary Table S13). The final results showed that 12 *CmGRAS* genes (*CmGRAS10*, *CmGRAS11*, *CmGRAS12*, *CmGRAS13*, *CmGRAS14*, *CmGRAS15*, *CmGRAS16*, *CmGRAS27*, *CmGRAS28*, *CmGRAS29*, *CmGRAS32*, *CmGRAS33*) were identified as from tandem, which all distributed in Chr3 and Chr6. Interestingly, Chr3 had two clusters, and Chr6 had a cluster containing 9 *CmGRAS* genes, suggesting the hot spots of *GRAS* gene distribution (Figure 1A). In addition, the number of *CmGRAS* genes considered as dispersed, proximal, WGD, segmental duplication was 21, 7, 6, 2. Interestingly, all *CmGRAS* genes were not considered singletons. These results indicated that tandem was the main driving force to promote the expansion of *CmGRAS* genes. We also calculated the Non-synonymous (*Ka*)/synonymous substitution sites (*Ks*) values of homologous gene pairs to analyze their election intensity and direction (Supplementary Table S14). All of homologous *CmGRAS* genes had *Ka*/*Ks* values less than 1, indicating that they were mainly subject to purification selection during the evolution process.

### *Cis*-acting elements in the promoter region of *CmGRASs*

**FIGURE 5:**
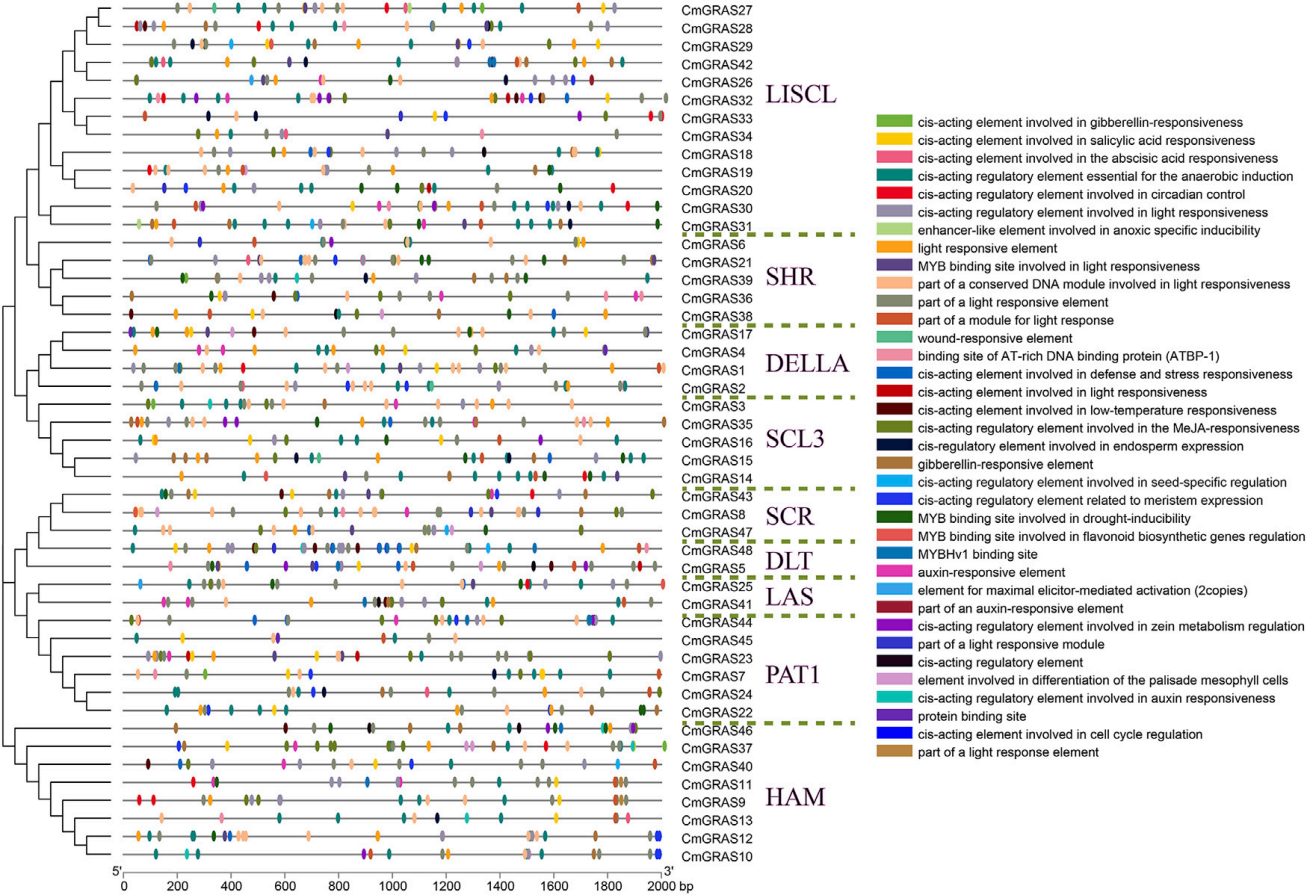
*Cis*-acting elements in the promoter region of *CmGRAS* genes. The left side dendrogram shows the phylogenetic tree of *CmGRAS* genes. The distribution of *cis*-acting elements in the 2,000 bp upstream promoter are shown. The different functions of *cis*-acting elements are represented by different colors, as shown on the right.

The *cis*-acting regulatory elements (CRE) in the promoter region were very important for comprehending the regulation of gene transcription. Here, we obtained 110–392 *cis*-acting elements in the 10,000 bp sequence upstream of the 48 *CmGRAS* genes using the PlantCare software (Supplementary Table S15). The results showed that there were a series of *cis*-acting regulatory elements in the promoter region of almost every *CmGRAS* member, and most of them played different roles in the plant life cycle. Obviously, visualization of all *cis*-acting elements predicted in all regions would make it difficult to observe, and we only visualized *cis*-acting elements in the 2,000 bp region upstream of the *CmGRAS* genes (Figure 5). The light responsive elements were widely distributed in PAT1 and SCL3 subfamily members, as has been widely reported in this area (Bolle et al., 2000; Torres-Galea et al., 2006). The *cis*-acting regulatory elements generally considered important were distributed in most *CmGRAS* genes, such as auxin responsive elements (AuR) and gibberellin responsive elements (GRE), which were considered to be related to flowering-related traits (Ayoub Khan et al., 2022). The genes (*CmGRAS8*, *CmGRAS18*, *CmGRAS26*, *CmGRAS42* and *CmGRAS43*) screened by collinearity analysis based on gene balance hypothesis had more regulatory elements (Figures 3B–E; Supplementary Table S15). In addition, a large number of response elements involved in abiotic stress were identified, such as low-temperature responsive elements (LTR), drought-inducibility elements (MBS), flavonoid biosynthetic gene regulators (MBS I), defense and stress responsive elements (TC-rich repeats), salicylic acid responsive elements (TCA-element), and wound-responsive elements (WUN-motif).

### Expression analysis of *CmGRAS* genes

**FIGURE 6:**
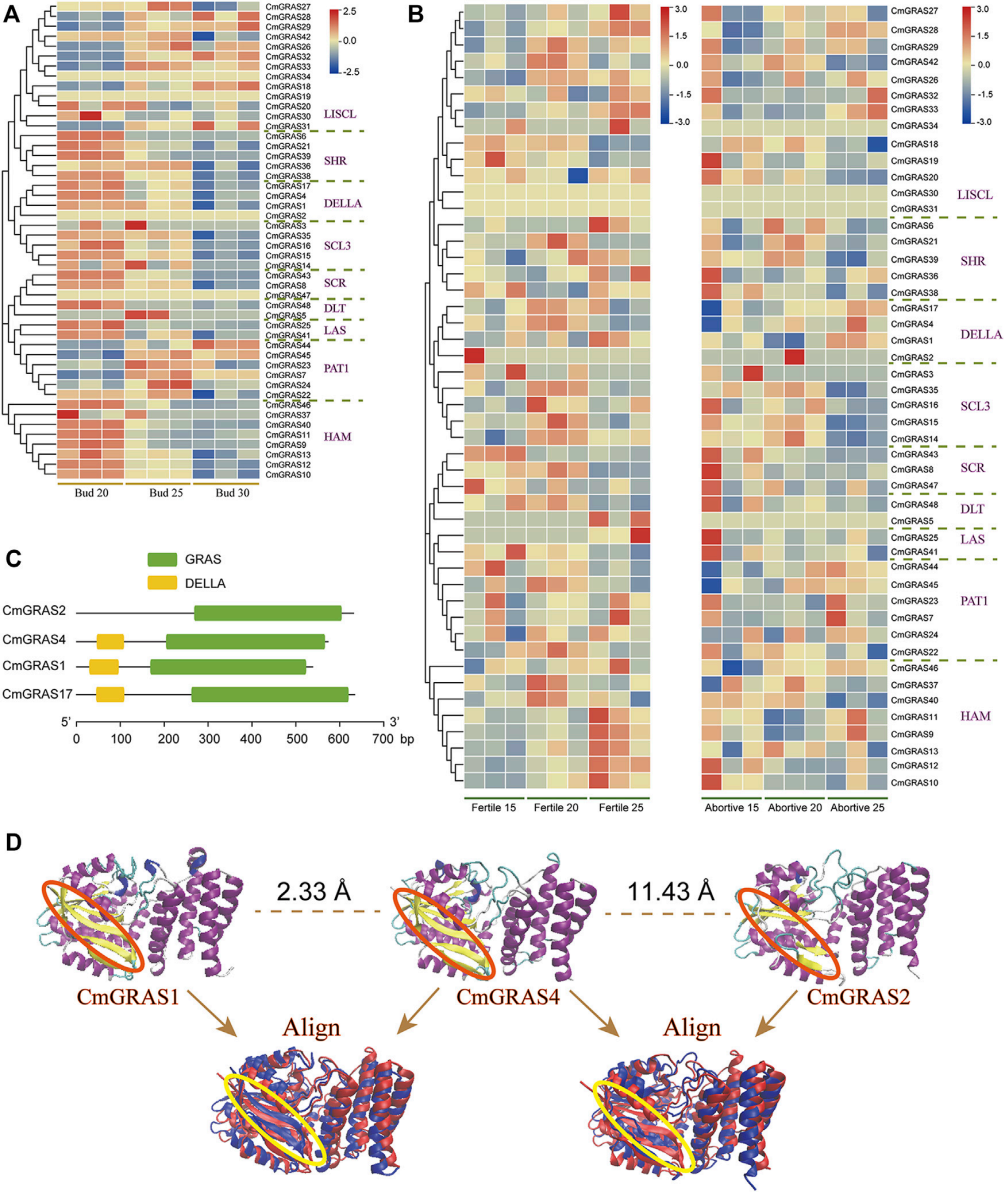
Transcriptome analysis of *CmGRAS* genes and protein three-dimensional structure of members in DELLA subfamily. **(A)** Heatmap of *CmGRAS* genes expression in Chinese chestnut buds 20, 25 and 30 days after flowering. The left side dendrogram shows the phylogenetic tree of CmGRAS genes. **(B)** Heatmap of *CmGRAS* genes expression in fertile and abortive ovules of Chinese chestnut buds at different stages (15-July, 20-July and 25-July). The left side dendrogram shows the phylogenetic tree of CmGRAS genes. **(C)** The conserved domain distribution of DELLA subfamily proteins. **(D)** Prediction of three-dimensional structure of DELLA subfamily proteins (by Swiss model) and the comparison of similarity.

The transcriptome data of three development stages of Chinese chestnut buds (20, 25 and 30 days after flowering) were analyzed to understand the expression pattern of *CmGRAS* genes (Figure 6A). Some *CmGRAS* genes showed a high expression consistent pattern at three stages of bud development, such as *CmGRAS24*, *CmGRAS41*, *CmGRAS44* and *CmGRAS45*, which suggested that they may play an important role in the growth of Chinese chestnut buds. Interestingly, all members of PAT1 subfamily showed high expression during the three stages, but the number of transcripts of members of other subfamilies differed greatly. For example, *CmGRAS2* of DELLA subfamily could hardly detect transcripts at three stages, while other members were continuously overexpressed. In the DELLA subfamily, only the *CmGRAS2* gene lacked the DELLA domain, which leads us to speculate that the deletion of the DELLA domain caused de functionalization of the *CmGRAS2* gene at the bud development stage. Although some *CmGRAS* members formed by tandem duplication had great differences in expression, they usually had similar expression patterns. For example, the expression of *CmGRAS10* and *CmGRAS11* gradually decreased, while the expression of *CmGRAS32* and *CmGRAS33* gradually increased with the development of buds, which may be caused by the fact that the members of genes from tandem duplication were located in closer positions and were easy to obtain more similar regulation.

In the transcriptome data of fertile and abortive ovules at different stages, there was significant differences in the expression of some genes in the two types of ovules (Figure 6B) (Du et al., 2021). For example, *CmGRAS48* in SHR subfamily maintained a high expression level at all three stages of fertile ovules, but its transcripts could hardly be detected in aborted ovules, which expression pattern also showed in *CmGRAS38*. Notably, these differences in gene expression levels persisted at the all three developmental stages and they may be related to the fertility of ovules. Interestingly, we still noticed that the *CmGRAS2* without DELLA domain in the DELLA subfamily had no transcript (TPM of all samples is less than 0.05) detected in both fertile and abortive ovules (Figures 6B,C). The three-dimensional structure of members of the DELLA subfamily were constructed to analysis other features beyond the DELLA domain that CmGRAS2 lacks (Figure 6D; Supplementary Figure S3; Supplementary Tables S16, S17), from which we can clearly find that CmGRAS2 lacks extended beta-strand (Figure 6D). Additionally, CmGRAS4 and CmGRAS1 with DELLA domain had very similar three-dimensional structures (RMSD = 2.33 Å) (Figure 6D), but was less similar to CmGRAS2 (RMSD = 11.43 Å). These structural differences could be detected more clearly by structure alignment, and the characterization of these structural differences in function needs further study.

## Discussion

GRAS transcription factors have been extensively studied due to its multiple functions in plant growth and development, resistance to biotic/abiotic stresses (Helariutta et al., 2000; Yuan et al., 2016; Shahan et al., 2022). At the same time, Chinese chestnut has been paid more and more attention, with the development of its nutritional value, medicinal value and economic value (Feng et al., 2018; Nie et al., 2021). In this study, we comprehensively characterized GRAS transcription factors in the whole-genome of Chinese chestnut for the first time, which enriches the resources for studying GRAS gene family and provides a reference for their research in Chinese chestnut, an important nut fruit tree.

In this study, 48 *GRAS* family genes were identified in Chinese chestnut, which is similar to the number of *GRAS* genes in pepper (*Capsicum annuum*) (50) (Liu et al., 2018) and castor bean (*Ricinus communis*) (48) (Xu et al., 2016) genomes, and more than *A. thaliana* (32) (Lee et al., 2008), but less than rice (57) (Tian et al., 2004), suggesting extensive duplication and diversification of GRAS family among species. The phylogenetic analysis divides the *CmGRAS* genes into nine subfamilies, which belong to the same subfamily has similar motif arrangement pattern and may have similar functions. Most *CmGRAS* genes contain fewer introns and this is consistent with previous speculation that *GRAS* genes in plants may originated from the ancient prokaryotes, because genes in prokaryotic genomes are usually intronless (Huang et al., 2015). *Cis*-acting elements involving multiple functions (such as responding to multiple hormones and stress) were identified in the promoter region of *CmGRAS* genes, which suggested that they have important functions in regulating the growth and development and responding to stress in Chinese chestnut. *CmGRAS* genes with more orthologous relationships with the reference species (*A. thaliana*, grape, oak and rice) had more *cis*-acting elements in the promoter region and showed a high expression pattern at different stages of bud and fertile/abortive ovules than other *CmGRAS* genes, implying that they are important in the evolution and function of the CmGRAS gene family. Tandem duplication is the main driving force for the expansion of the CmGRAS gene family, and they have similar expression patterns, which may be related to their relatively close spatial location and easy acceptance of similar regulation (Yu et al., 2021).

GRAS transcription factors play many roles in plant growth and development, and the study in plant meristem is an important aspect (Helariutta et al., 2000; Shahan et al., 2022). SCR was the first protein identified, isolated and purified in GRAS family, which is very specific expression in endothelial layer and its initial cell (stem cell), and helped the stem cells to be in an undifferentiated state (Di Laurenzio et al., 1996; Nakajima et al., 2001; Shahan et al., 2022). *AtLAS* is a key gene controlling the formation of axillary meristem in *A. thaliana*, and the mutant of *LAS* cannot form lateral branches (Greb et al., 2003). *MOC1* is a GRAS-like gene in rice and its overexpression can significantly increase the number of tillers, while the mutant cannot form axillary buds and tillers (Li et al., 2003). *RGA* regulates the growth of intermediate meristem, which can inhibit the expansion of leaves (Silverstone et al., 1998). In this study, some *CmGRAS* genes showed a high expression consistent pattern at three stages of bud development, such as *CmGRAS44*. Interestingly, auxin responsive elements (AuR) and *cis*-acting regulatory element related to meristem expression (CAT-box) were identified in the promoter region of *CmGRAS44* (Figure 5). Notably, auxin is involved in almost all aspects of plant growth and development, especially affecting the growth and development of bud and root tips (Woodward and Bartel, 2005). These results suggested that *CmGRAS44* may be related to auxin synthesis and affect the growth and development of Chinese chestnut buds. Actually, *GRAS* genes even showed opposite expression changes in buds and roots under auxin treatment, which suggested that *GARS* transcription factors regulate auxin signals through complex networks to affect gene expression (Huang et al., 2015).

In addition, a large number of studies had reported that members of GRAS gene family participate in the regulation of GA signal transduction (Gubler et al., 2002; Griffiths et al., 2006; Heo et al., 2011). For example, many members of DELLA subfamily in *A. thaliana* participate in the regulation of GA signals, such as GAI, RGA, RGL1, RGL2, and RGL3, and the conservative DELLA sequence they contain is necessary for GA signal sensing (Hirsch and Oldroyd, 2009). In the flower regulation network, GA is crucial to the development of reproductive organs, especially to the sex determination of flowers (Song et al., 2013; Plackett et al., 2014). As a monoecious plant, the proportion of female and male flowers in Chinese chestnut can reach 1:2400–4000, and the low number of female flowers and excessive consumption of tree nutrition by abundant male flowers are significant causes for limiting the yield of Chinese chestnut (Guo et al., 2012; Chen et al., 2019). In this study, there are significant differences in transcript abundance of some *CmGRAS* genes in transcriptome data of fertile and abortive ovules, such as *CmGRAS48* and *CmGRAS38*. These genes were highly expressed in all growth stages of fertile ovules, while transcripts were almost undetectable in abortive ovules. In addition, the transcripts of some *CmGRAS* genes decreased (*CmGRAS41*) or increased (*CmGRAS9*) sharply in the three developmental stages of the fertile ovule, indicating their potential active function in the ovule development process. Considering the gibberellin responsive elements (GRE) (Figure 5) detected in their promoter regions and the relationship between *GRAS* genes and GA signal transduction (Huang et al., 2015), we speculate that they may be related to the fertility of ovules and involve in mediating GA responses during the development of Chinese chestnut buds. In addition, we can hardly detect the transcript of *CmGRAS2* in all samples in this study and it is a member without DELLA domain in DELLA subfamily. Notably, the same result also appeared many tissues (roots, stems, leaves, fruits) of other plants in our previous studies (Yu et al., 2021), that is, the DELLA subfamily members without DELLA domain could hardly detect the expression. CmGRAS2 protein had poor similarity with other members of DELLA subfamily in three-dimensional structure, and lacked extended beta-strand and other secondary structures. These results spur us to speculate that these members without DELLA domain became pseudogenes, and it may be due to the loss of DELLA domain that they were de functionalized. The specific reasons for these members without DELLA domain in DELLA subfamily almost not expressing in multiple tissues of different species need to be further explored. Of cause, these analysis results were based on current genome and protein sequence annotation information, and the continuously improved genome and protein annotation of Chinese chestnut may be helpful to understand these results.

The functions of *GRAS* genes in plants are diversified, such as GA signal transduction regulation, meristem growth and stress resistance, which are all related to the current factors limiting the yield of Chinese chestnut (such as imbalance of male and female flowers, drought stress) (Chen et al., 2019). Focusing on the multiple functions of *GRAS* genes in plants, it is very valuable to carry out relevant research on *GRAS* gene in Chinese chestnut. In this study, the analysis of physicochemical properties, phylogenetic analysis, motif distribution, gene structures, duplication model and *cis*-acting elements of 48 *CmGRAS* genes had systematically characterized this important gene family in Chinese chestnut. The transcriptome expression profile showed that the CmGRAS gene family had important function in Chinese chestnut bud development and ovule fertility. Our analysis provides a basis for clarifying the evolution and function of the *CmGRAS* genes and provide fundamental information about the GRAS family in Chinese chestnut.

## Data Availability

The original contributions presented in the study are included in the article/Supplementary Material, further inquiries can be directed to the corresponding author.

## Supplementary material

SUPPLEMENTARY FIGURE S1 The phylogenetic tree of GRAS genes in Chinese chestnut, *Arabidopsis thaliana* and rice was constructed by neighbor-joining method.

SUPPLEMENTARY FIGURE S2 Normal Distribution of *Ks* Values of Collinear Genes in *Castanea mollissima*.

SUPPLEMENTARY FIGURE S3 Prediction of three-dimensional structure of DELLA subfamily proteins (by AlphaFold 2) and the comparison of similarity.
