# Supplementary figures and images for: Genome-wide identification, evolution and transcriptome analysis of GRAS gene family in Chinese chestnut (Castanea mollissima)
Source: Front Genet. 2023 Jan 4;13:1080759. doi: 10.3389/fgene.2022.1080759 (PMC9845266; doi:10.3389/fgene.2022.1080759)

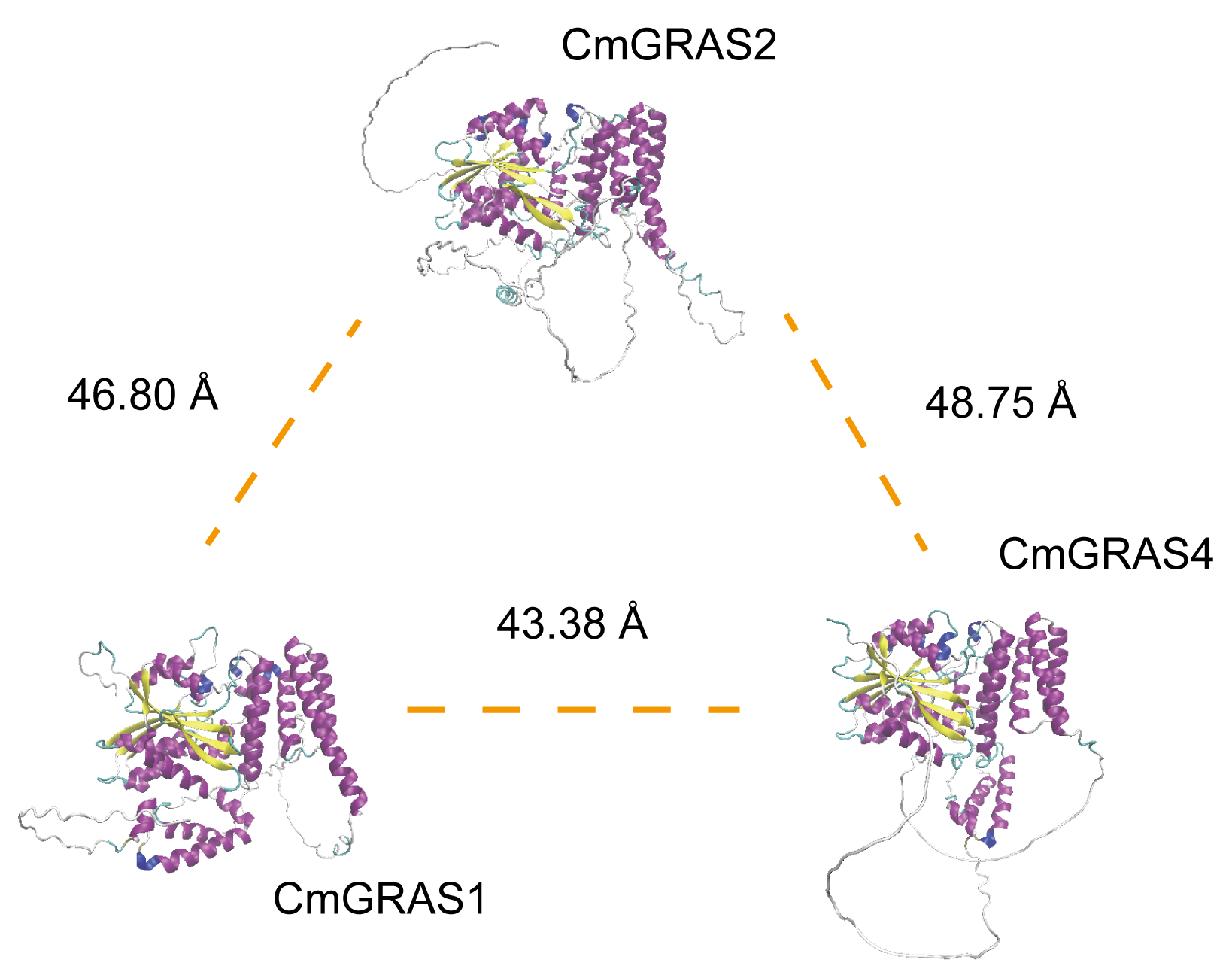

Supplement: Supplementary file 1 [file Image3.TIF]

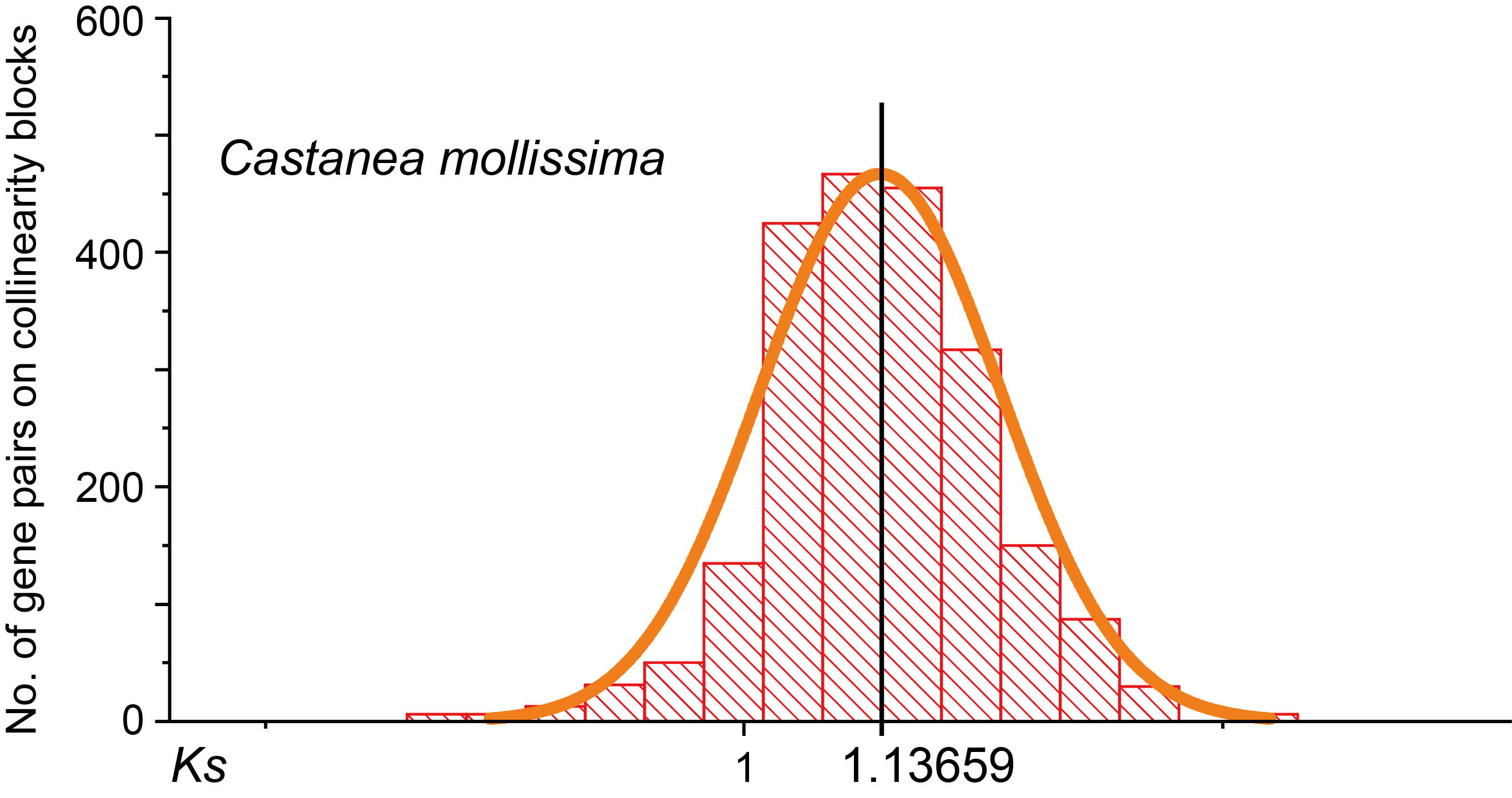

Supplement: Supplementary file 2 [file Image2.TIF]

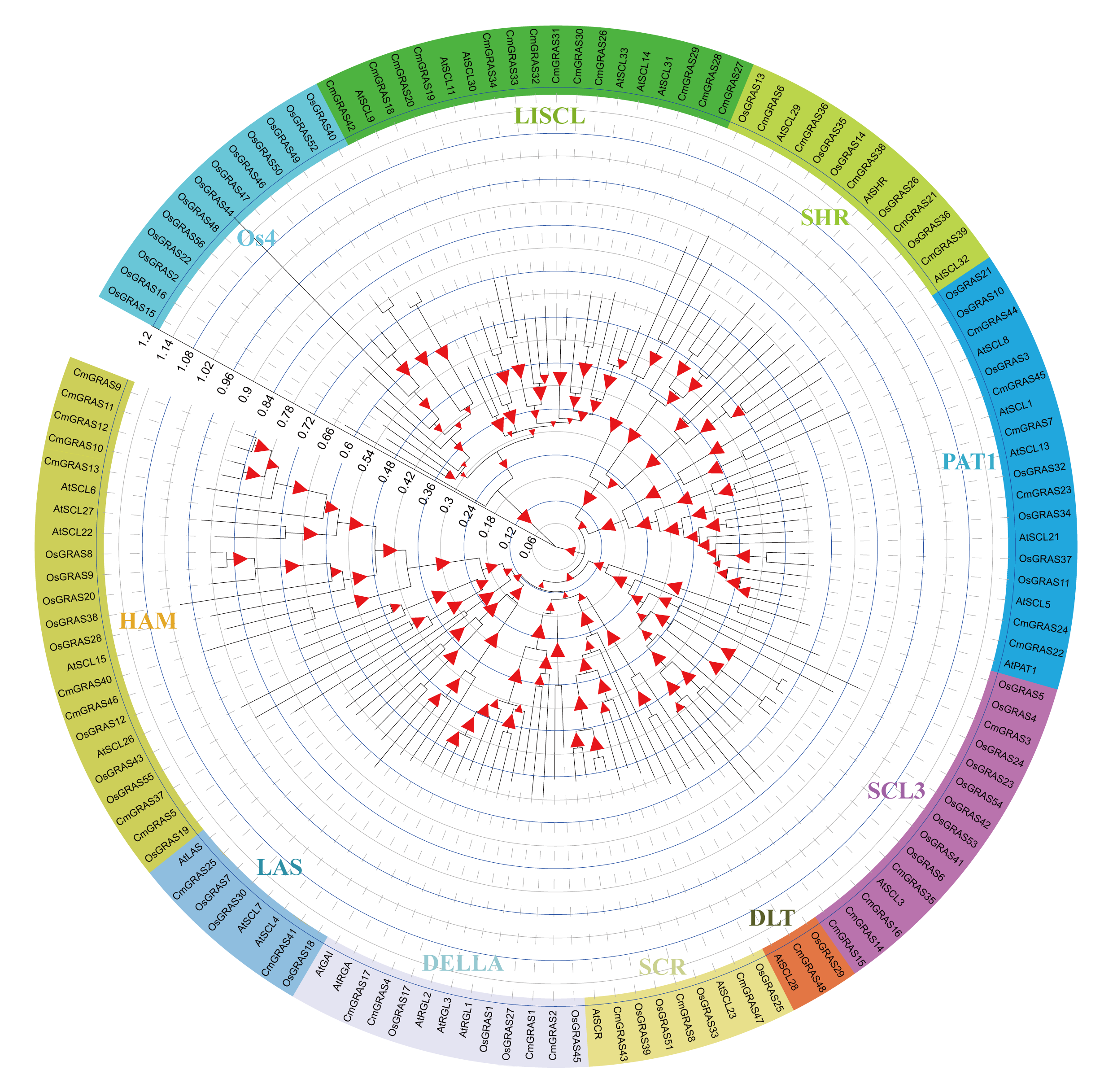

Supplement: Supplementary file 3 [file Image1.TIF]
